# Endotracheal cuff undersizing diagnosed by computed tomography: Case report

**DOI:** 10.1002/ccr3.5193

**Published:** 2021-12-26

**Authors:** Hong‐Lei Wu, Yan‐Man Zhang, Jia‐hai Shi, Pei‐pei Ji, Wang‐Qin Shen

**Affiliations:** ^1^ Nursing Department Nantong University and Affiliated Hospital of Nantong University Nantong City China; ^2^ Department of Imageology Affiliated Hospital of Nantong University Nantong City China; ^3^ Department of Cardiothoracic Surgery Affiliated Hospital of Nantong University Nantong City China; ^4^ Nursing Department Nantong University Nantong City China

**Keywords:** complications, endotracheal tube confirmation, maximum leak pressure, ventilator management

## Abstract

We herein report a case in which the trachea could be completely sealed only when the cuff pressure reached 100 cmH_2_O. An excessive cross‐sectional area of the trachea is a rare phenomenon, but we believe that our case will be helpful for clinicians who encounter similar situations.

## Graphical abstract

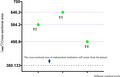

## 1 INTRODUCTION

Endotracheal tube cuffs create a seal between the endotracheal tube and the trachea, preventing aspiration of fluids and pathogens from the pharynx to the lungs and ventilation leaks. Consensus suggests that the cuff pressure of endotracheal tubes should range from 20 to 30 cmH_2_O.^1^ In clinical practice, however, cuff pressures of >30 cmH_2_O may be required to create a seal in the trachea.^2^ One possible reason for this is that the cuff size may be unsuitable.

Global guidelines currently lack clear guidance or consensus on selection of the most appropriate oral endotracheal tube model.^3^ Studies outside China suggest that an endotracheal tube with an inner diameter (ID) of 7.0–8.0 mm is usually chosen for women and that a tube with an ID of 8.0–8.5 mm is usually chosen for men. A formula based on age is traditionally used to predict the most appropriate size of endotracheal tube for children. For example, Cole's formula^4^ for an endotracheal tube without the cuff is ID in mm = (0.25 × age in years) +4.0, and Motoyama's formula^5^ for an endotracheal tube with the cuff is ID in mm = (0.25 × age in years) +3.5. Thus, there is no consensus on the choice of endotracheal tubes for patients, and an accurate and feasible method is urgently needed.

## 2 CASE PRESENTATION

A 70‐year‐old woman was referred to our hospital because lung cancer had been found by chest X‐ray computed tomography (CT) in October 2019. Chest CT showed right lower lung cancer with mediastinal and right hilar lymph node metastasis, multiple small nodules in the right lower lung, chronic bronchitis, emphysema, and multiple bullae in both lungs. Her height was 158 cm, and her weight was 50 kg. Her blood pressure was 169/99 mmHg, and her heart rate was 65 bpm. The patient underwent lobectomy and lymph node dissection via thoracoscopy in October 2019. She received endotracheal intubation and ventilator‐assisted respiration after the operation in the thoracic and cardiac surgery intensive care unit. Her cuff pressure was maintained with the minimum leak technique and measured with a cuff pressure gauge.^6^ Subsequent cuff manometer measurement demonstrated a pressure of 100 cmH_2_O.

## 3 RADIOGRAPHIC IMAGING

**FIGURE 1:**
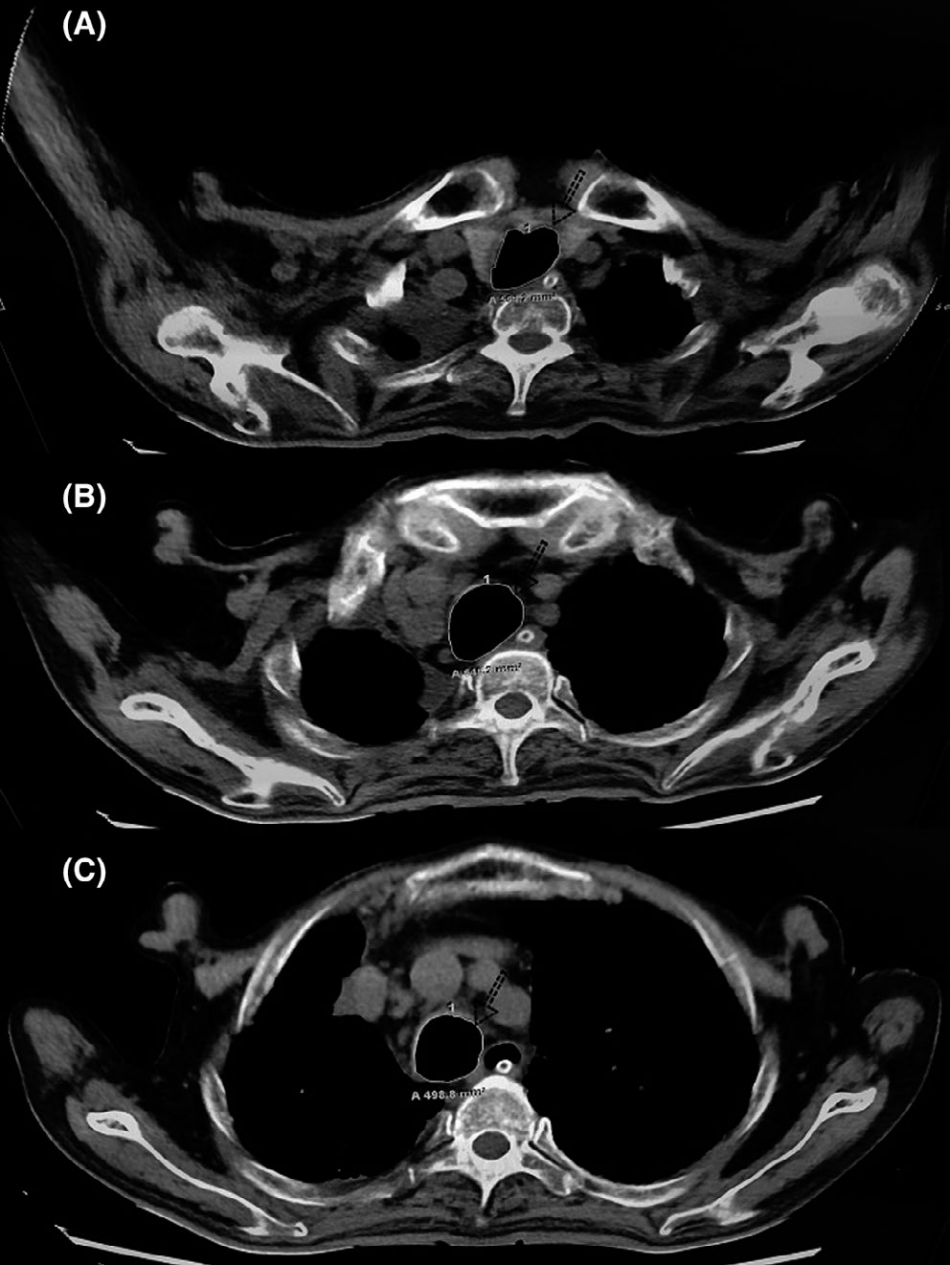
Computed tomography images used for measurement of tracheal cross‐sectional area at different upper vertebral levels. (A) T2 vertebra (584.2 mm^2^). (B) T3 vertebra (646.2 mm^2^). (C) T4 vertebra (498.8 mm^2^)

**FIGURE 2:**
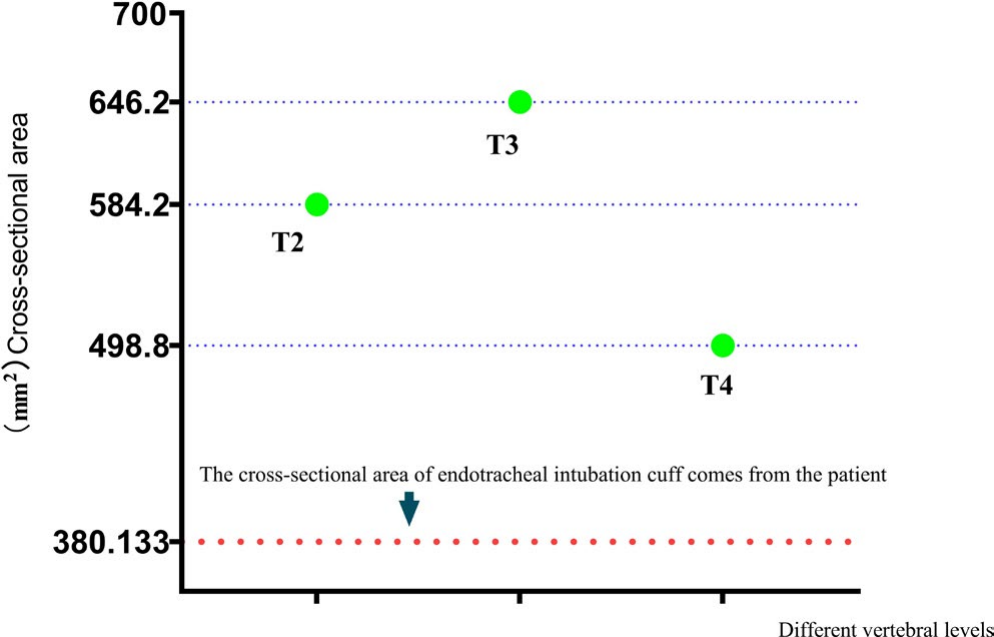
Comparison of cross‐sectional area of endotracheal tube cuff and patient's trachea at T2, T3, and T4 vertebra

Using the patient's tracheal cross‐sectional area at different vertebrae, endotracheal tube cuff undersizing was diagnosed by chest CT. The endotracheal tube was inserted through the mouth, and CT showed that the tracheal area at the T2 vertebra was 584.2 mm^2^ (Figure 1A), that at the T3 vertebra was 646.2 mm^2^ (Figure 1B), and that at the T4 vertebra was 498.8 mm^2^ (Figure 1C). The ID of the endotracheal tube was 7.0 mm (Guangzhou Weili Medical Equipment Co., Ltd.), the cuff diameter was 22 mm, and the cuff cross‐sectional area was 380.13 mm^2^. The patient's tracheal cross‐sectional area was greater than the cross‐sectional area of the cuff (380.13 mm^2^) (Figure 2).

## 4 DISCUSSION

Aljatlany et al.^7^ introduced the following formula for measuring the cross‐sectional area of the trachea: Cross‐sectional area = −171.834 + (0.5850 × age in years) + (86.8685 × sex) + (2.3953 × height in cm), where sex is denoted by “1” for men and “0” for women). The final calculated tracheal cross‐sectional area of the patient in the present case was 247.5734 mm^2^. According to the height‐based nomogram for endotracheal tube size selection derived from the CT‐imaging study by Cao et al.,^8^ the final predicted endotracheal tube size of the patient was 6.5 or 7.0 mm. Theoretically, according to the above two selection methods, the cuff of the endotracheal tube can effectively seal the airway when the intracuff pressure is 30 cmH_2_O. However, the true tracheal cross‐sectional area of the patient in the present case was unusual.

The cuff seals the airway during mechanical ventilation. An endotracheal cuff pressure of 20–30 cmH_2_O is adequate for most patients, but lack of a tracheal seal still occurs in a small number of patients.^9^, ^10^ The airway of the patient in our case could be completely sealed only when the cuff pressure of the endotracheal tube reached 100 cmH_2_O. The diagnosis of cuff undersizing was made by chest X‐ray CT. Our main finding was that the actual cross‐sectional area of the airway was significantly larger than the cross‐sectional area of the cuff. The real cross‐sectional area of the airway in our patient obviously exceeded that of the general population. Thus, this case illustrates the importance of rapid diagnosis of undersizing by chest CT to prevent mismatch between the cuff and airway to ensure that the selected endotracheal tube matches the patient's airway.

Consensus suggests that the cuff pressure of the endotracheal tube should range from 20 to 30 cmH_2_O.^1^ Excessively high or low cuff pressures have been associated with complications such as tracheal stenosis, air leakage, microaspiration of secretions, and ventilator‐associated pneumonia.^11^ As shown in Figure 1, the cross‐sectional area of the trachea was significantly larger than the cross‐sectional area of the cuff; thus, only when the cuff pressure was 100 cmH_2_O could the cuff seal the airway. According to expert consensus, such patients are prone to ventilator‐associated pneumonia.

## 5 CONCLUSION

The cross‐sectional area of the trachea should be periodically evaluated by chest CT during anesthesia to assure that mismatch is not present. When the intracuff pressure is 20–30 cmH_2_O, some patients' trachea cannot be effectively sealed.

## CONFLICTS OF INTERESTS

The authors declare that they have no competing interests.

## AUTHOR CONTRIBUTION

Hong‐Lei Wu and Pei‐pei Ji: were actively involved in the clinical care of the patient. Hong‐Lei Wu and Yan‐Man Zhang: wrote the manuscript. Jia‐hai Shi and Wang‐Qin Shen: revised the manuscript.

## ETHICAL APPROVAL

This case report is a small part of our big study. All patients provided informed consent for inclusion before they participated in the study. The study was registered with the Chinese Clinical Trial Registry (ChiCTR‐COC‐15006459) on 29 May 2015.

## CONSENT

Written informed consent was obtained from the patient for publication of this case report and any accompanying images. A copy of the written consent is available for review by the editor in chief of this journal.

## Data Availability

The data that support the findings of this study are available from the corresponding author upon reasonable request.
